# Clinical and radiological outcomes of non-window-type bioactive glass–ceramic cage in single-level ACDF versus PEEK cage filled with autologous bone

**DOI:** 10.1038/s41598-024-54786-3

**Published:** 2024-02-19

**Authors:** Ji-Won Kwon, Yong Ho Lee, Byung Ho Lee, Jae Hong Kim, Kyung Soo Suk

**Affiliations:** 1 Department of Orthopedic Surgery, Yonsei University College of Medicine, Eonju-ro 63-gil, Gangnam-gu, Seoul, 06229 Republic of Korea; 2 Department of Orthopedic Surgery, Yonsei Baro-Chuk Hospital, Seoul, Republic of Korea

**Keywords:** Anterior cervical discectomy and fusion (ACDF), Bioactive glass–ceramic, Bone graft substitute, PEEK, Autologous iliac bone graft, Implants, Outcomes research

## Abstract

Bioactive glass–ceramic (BGC) cage is a substitute for polyether ether ketone (PEEK) cages in anterior cervical discectomy and fusion (ACDF). Only a few comparative studies exist using PEEK and non-window-type BGC cages (CaO–SiO_2_–P_2_O_5_–B_2_O_3_) in single-level ACDF. This study compared PEEK cages filled with autologous iliac bone grafts and BGC cages regarding clinical safety and effectiveness. A retrospective case series was performed on 40 patients who underwent single-level ACDF between October 2020 and July 2021 by a single orthopedic spine surgeon. The spacers used in each ACDF were a PEEK cage with a void filled with an autologous iliac bone graft and a non-window-type BGC cage in 20 cases. The grafts were compared pre-operatively and post-operatively at 6 weeks and 3, 6, and 12 months. Post-operative complications were investigated in each group. Clinical outcome was measured, including Visual Analog Scale (VAS) scores of neck and arm pains, Japanese Orthopedic Association score (JOA), and Neck Disability Index (NDI). Dynamic lateral radiographs were used to assess the inter-spinous motion (ISM) between the fusion segment and subsidence. The fusion status was evaluated using a computed tomography (CT) scan. Overall, 39 patients (19 and 20 patients in the PEEK and BGC groups, respectively) were recruited. Eighteen (94.7%) and 19 (95.0%) patients in the PEEK and BGC groups, respectively, were fused 12 months post-operatively, as assessed by ISM in dynamic lateral radiograph and bone bridging formation proven in CT scan. The PEEK and BGC groups showed substantial improvement in neck and arm VAS, JOA, and NDI scores. No substantial difference was found in clinical and radiological outcomes between the PEEK and BGC groups. However, the operation time was considerably shorter in the BGC group than in the PEEK group. In conclusion, a non-window-type BCG cage is a feasible substitute for a PEEK cage with an autologous iliac bone graft in single-level ACDF.

## Introduction

Anterior cervical discectomy and fusion (ACDF) have been the foremost treatment option for degenerative cervical spine disease since they were introduced by Smith and Robinson in the 1950s^1–3^. Neural elements in ACDF can be decompressed in two ways. Direct decompression involves the resection of herniated disc material, impinging bone, or ligament around neural foramina, whereas indirect decompression is achieved by inserting a graft into the removed disc space to restore disc height, neural foramina, and normal segmental lordosis. Therefore, to achieve a satisfactory clinical outcome, proper graft insertion is crucial to maintain the indirect decompression effect until fusion^3,4^. Traditionally, the autologous tri-cortical iliac bone graft is considered the gold standard for graft selection. However, possible donor-site morbidities, such as iliac bone fracture, hematoma, infection, increased blood loss, and operation time, exist^5,6^; therefore, operators have attempted to uncover new graft spacer substitutes, and several alternative grafts have been introduced, including titanium, polyether ether ketone (PEEK), and structural allograft bone cages^5,7^. Favorable clinical outcomes and higher fusion rates have been reported with these graft materials. However, subsidence, graft breakage, and fusion failure attributed to the graft’s innate physical property and biocompatibility remain issues yet to be addressed^8^. Since the discovery of bioactive glasses (BG), numerous trials have been conducted to make BG for implant material to bond living tissue^9,10^. The well-known 45S5 Bioglass^®^ was invented following the testing of numerous compositions^11–13^. It is known for making a bond with host bone by making a hydroxyapatite layer after contact with biological fluids *in vivo*^14^. Bioactive glass–ceramic (BGC) is one of the derivatives of BG and can induce bone integration with a hydroxyapatite-coated layer on its surface^15^. Therefore, it would be an alternative to the abovementioned cages, and we can expect a favorable clinical and radiological outcome^11,13,15^. Recently, a report on the BGC (CaO–SiO_2_–P_2_O_5_–B_2_O_3_) spacer in terms of its modulus of elasticity and contact area using mechanical tests and finite element analysis exists^16^. However, the main argument was the BGC cage’s superiority in terms of larger contact area and better subsidence performance, and determining whether this is correlated with actual clinical outcomes is necessary. Therefore, this retrospective case series study aimed to evaluate the comparison of the safety and effectiveness of a BGC cage with a PEEK cage filled with autologous iliac bone graft in single-level ACDF.

## Methods

### Patient population

Forty consecutive patients underwent single-level ACDF between October 2020 and July 2021 by a single spine surgeon with at least 20 years of post-fellowship surgical experience in a single center. The inclusion criteria were patients aged between 19 and 75 years with symptoms of radiating pain or myelopathy or both, which are the indications for single-level ACDF at the C3-7 vertebral body. The senior surgeon performed only the ACDF using the BGC procedure from October 2020 to January 2021 to compare ACDF with the BGC cage and PEEK cage filled with autologous bone, which minimized the randomization issue. After determining that patients had been secured over 12 months, the senior surgeon performed only ACDF with a PEEK cage filled with autologous bone for the remaining study period with a prospective plan. Patients were followed up for 12 months post-operatively to evaluate the clinical and radiological outcomes. The exclusion criteria included evidence of systemic or locally relevant cervical spine infection or a medical condition that requires medication, including steroids or nonsteroidal anti-inflammatory drugs that could affect fusion and interfere with the outcome. The patient’s smoking status was also investigated. Post-operative adverse effects, including dysphagia and hematoma, were investigated. This retrospective study was approved by the Institutional Review Board (Yonsei University Institutional Review Board and Ethics Committee: 2022-0801-001), which issued a waiver regarding the need for informed consent. All methods were performed in accordance with the relevant guidelines and regulations.

### Graft profile

**Figure 1:**
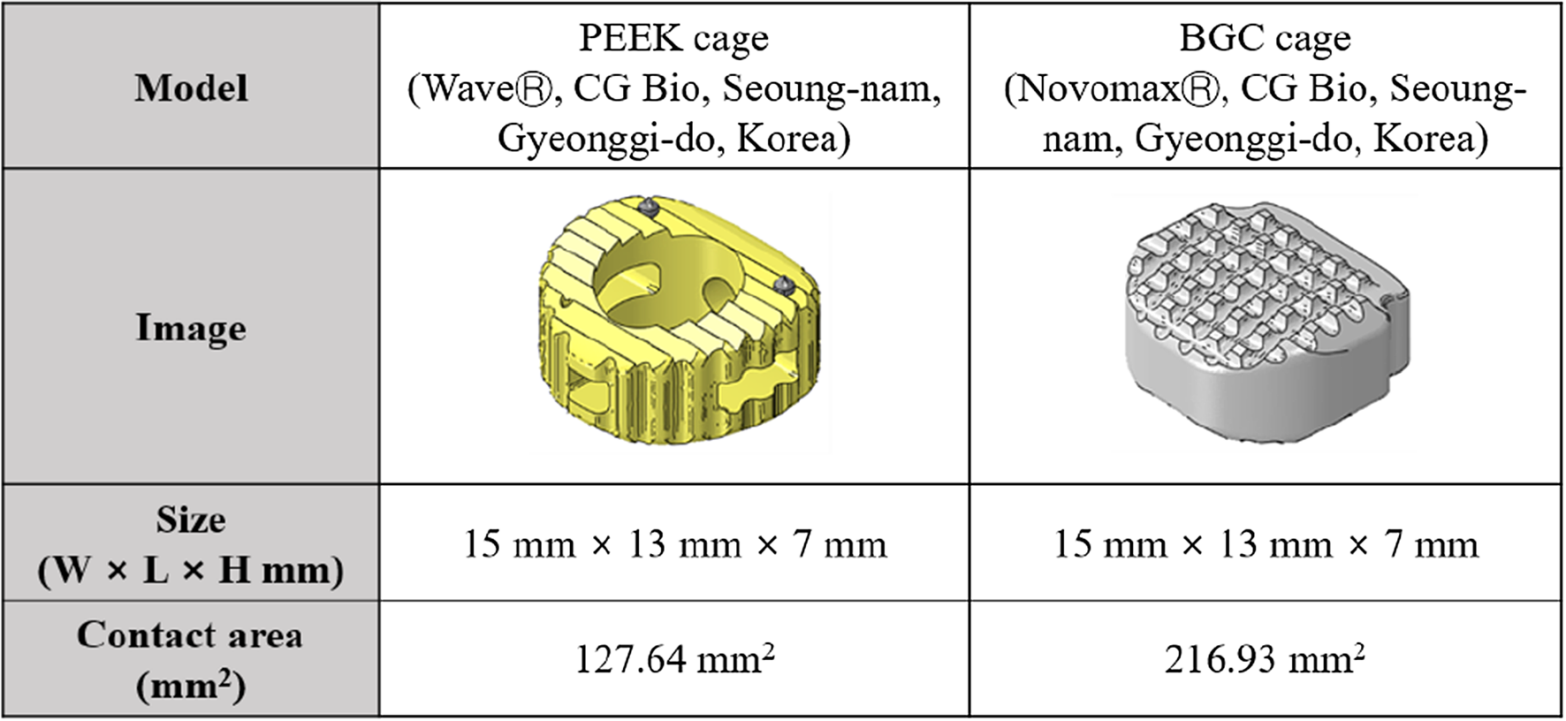
Visualization of cage model, approximate appearance, size and contract area related to PEEK cage and non-window type BGC cage.

The spacers used in each ACDF were PEEK cage (Wave^®^, CG Bio, Seoung-nam, Gyeonggi-do, Korea) filled with autologous iliac bone graft and BGC cage (Novomax^®^, CG Bio, Seoung-nam, Gyeonggi-do, Korea)^17^. The composition of the BGC cage used in this study was CaO–SiO_2_–P_2_O_5_–B_2_O_3_ (named BGC-7). It had no void to be filled with autologous or allogenic bone material. The materials used were approved by the Food and Drug Administration (14–592), where this study was conducted^11^. Additionally, the size was specified as 15 × 13 × 7 mm in standard size for the PEEK and BGC cages (Fig. 1).

### Clinical evaluation

The grafts were compared pre-operatively and post-operatively at 6 weeks and 3, 6, and 12 months. Clinical outcome was evaluated using the VAS score of neck and arm pains^18^, JOA score^19^, and NDI^20,21^.

### Radiological evaluation

**Figure 2:**
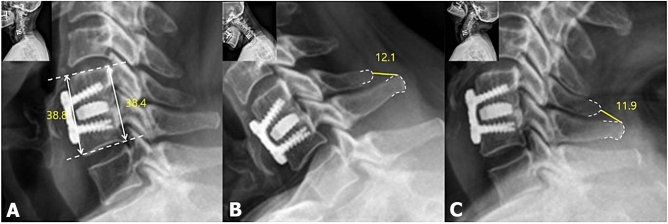
12 months postoperative dynamic lateral radiograph of a 66 years-old woman. Length of anterior and posterior border of fusion segment was measured (**A**). If there is 3 mm or more shorten is calculated in difference with mean of anterior or posterior border between preoperative and postoperative 12 months, defined as subsidence. Inter-spinous motion (ISM) is measured between most prominent point of spinous process within fusion segment in (**B**) Flexion and (**C**) Extension view. if there is > 1 mm difference, was defined as pseudoarthrosis.

**Figure 3:**
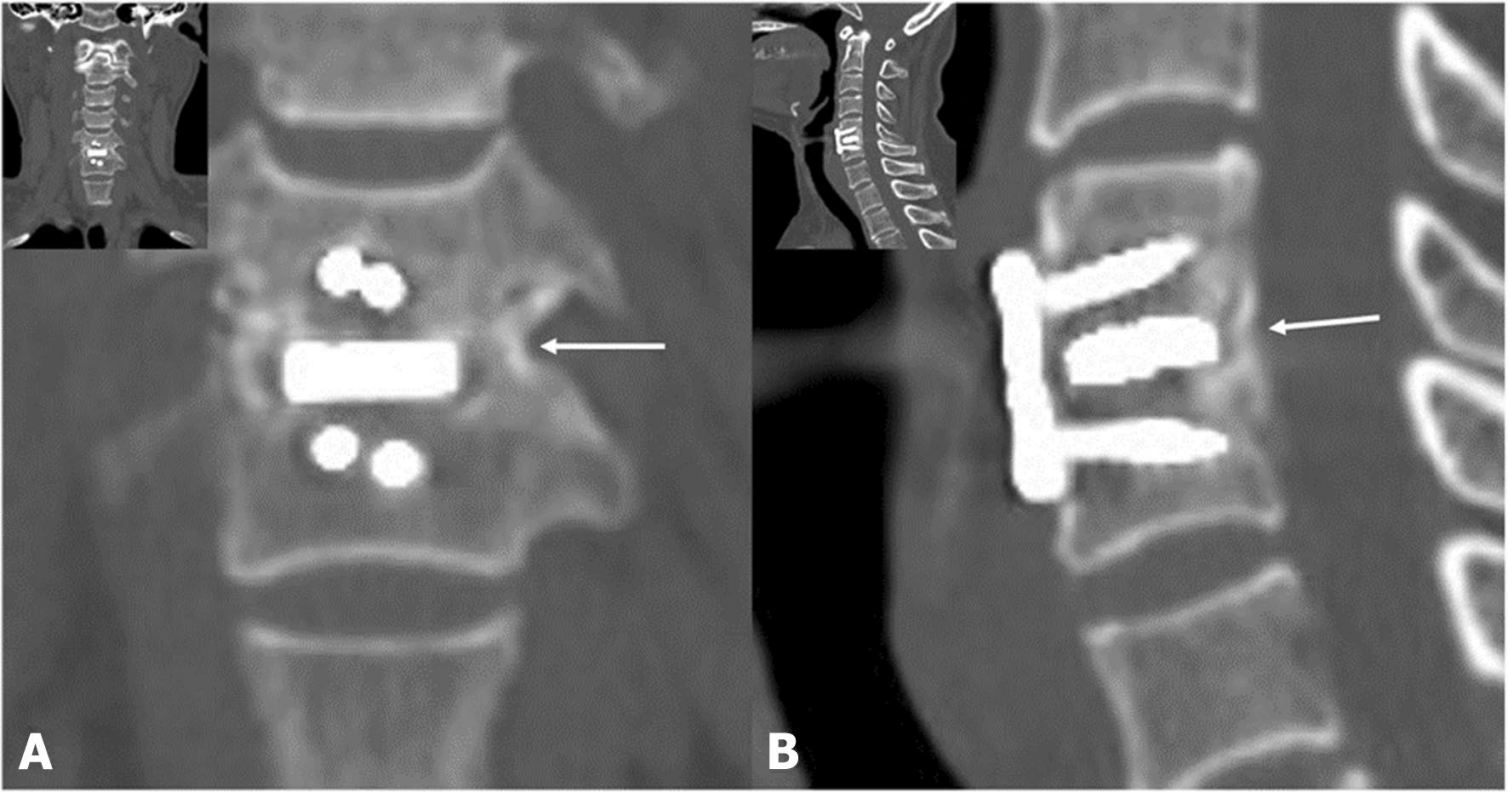
12 months postoperative CT image. Bone bridge formation (white arrows) was assessed in coronal (**A**) and sagittal (**B**).

Dynamic lateral radiographs were used to assess ISM in a 150% magnified view between the most prominent point of the spinous process in the fusion segment. More than 1 mm change of ISM between 6 weeks and 12 months simultaneously post-operatively was regarded as pseudoarthrosis^22^. In the lateral radiograph, cage subsidence was defined as > 3 mm loss at the one-level segment heights, which were calculated as the mean anterior and posterior vertebral body heights between the upper and lower margins of the upper and lower vertebral bodies, respectively, at the fused segment between immediate post-operative and post-operative 12 months (Fig. 2)^23^. A CT scan was used to evaluate the fusion status, which is defined as bone bridging formation around graft material in the fusion segment 12 months post-operatively (Fig. 3). Fusion status was assessed based on the agreement of two orthopedic surgeons with 5 and 8 years of experience who were not involved in the treatment. Inter- and intra-observer agreements were assessed using Cohen’s kappa value (95% confidence interval) according to Landis et al.’s method^24^. Two reviewers analyzed the CT scans and dynamic radiographs for bone bridging formation around the cage and ISM after a 3-week interval to investigate the intra-rater agreement. Disagreements in the radiographic results for assessment of fusion status were resolved through discussion between reviewers with a unanimous decision. All radiographic parameters were measured using an internal caliper tool in the software (Centricity 3.0, General Electric Medical System, Milwaukee, WI, USA).

### Surgical technique and post-operative management

Intervertebral disc and endplate cartilage were removed with a cervical curette and high-speed burr after the typical left-sided anterior Smith–Robinson method for soft tissue dissection. Intervertebral disc space was diverted while the disc was being released using the Caspar cervical pin distraction system. Next, the remaining disc, posterior longitudinal ligament complex with or without ossification, and compressive radial osteophyte around the nerve root were removed using a Kerrison punch to achieve the greatest amount of decompression. Excision of the uncinate process using an ultrasonic scalpel was added if the degeneration of the uncovertebral joint was considerably severe to compress the nerve root and impossible to extract with a manual punch grip. After inserting the cage trial, the cage size was selected, and the fusion bed was subsequently prepared to meet the graft’s contour to facilitate fusion. In the PEEK group, additional autologous iliac bone was harvested from the left anterior superior iliac spine. Square-shaped cortical bone osteotomy was performed to create a window after making an incision of < 1 cm at a point 2 cm above the ASIS, and cancellous bone was subsequently harvested using a gauge. Except for the additional autologous iliac bone harvesting operation to fill a void in a PEEK cage, the cage insertion processes were identical for each PEEK or BGC cage. The standard size was specified as 15 × 13 × 7 mm size as follows. The cage height can be affected by pre-operative/intra-operative findings; however, direct decompression could be achieved through uncinate process resection; therefore, the surgeon attempted to unify the cage specifications as much as possible. Furthermore, to ensure the greatest bone bridge formation, any bone dust created by grinding osteophyte with a high-speed burr was collected and placed around the graft material. In every instance, an anterior plating system (VENTURE^®^, Medtronic, MN, US) was used after cage implantation. Post-operatively, the post-operative drain was left in for 2 days before being removed. All patients in the two groups were provided a rigid neck collar 12 weeks post-operatively.

### Statistical analysis

**Figure 4:**
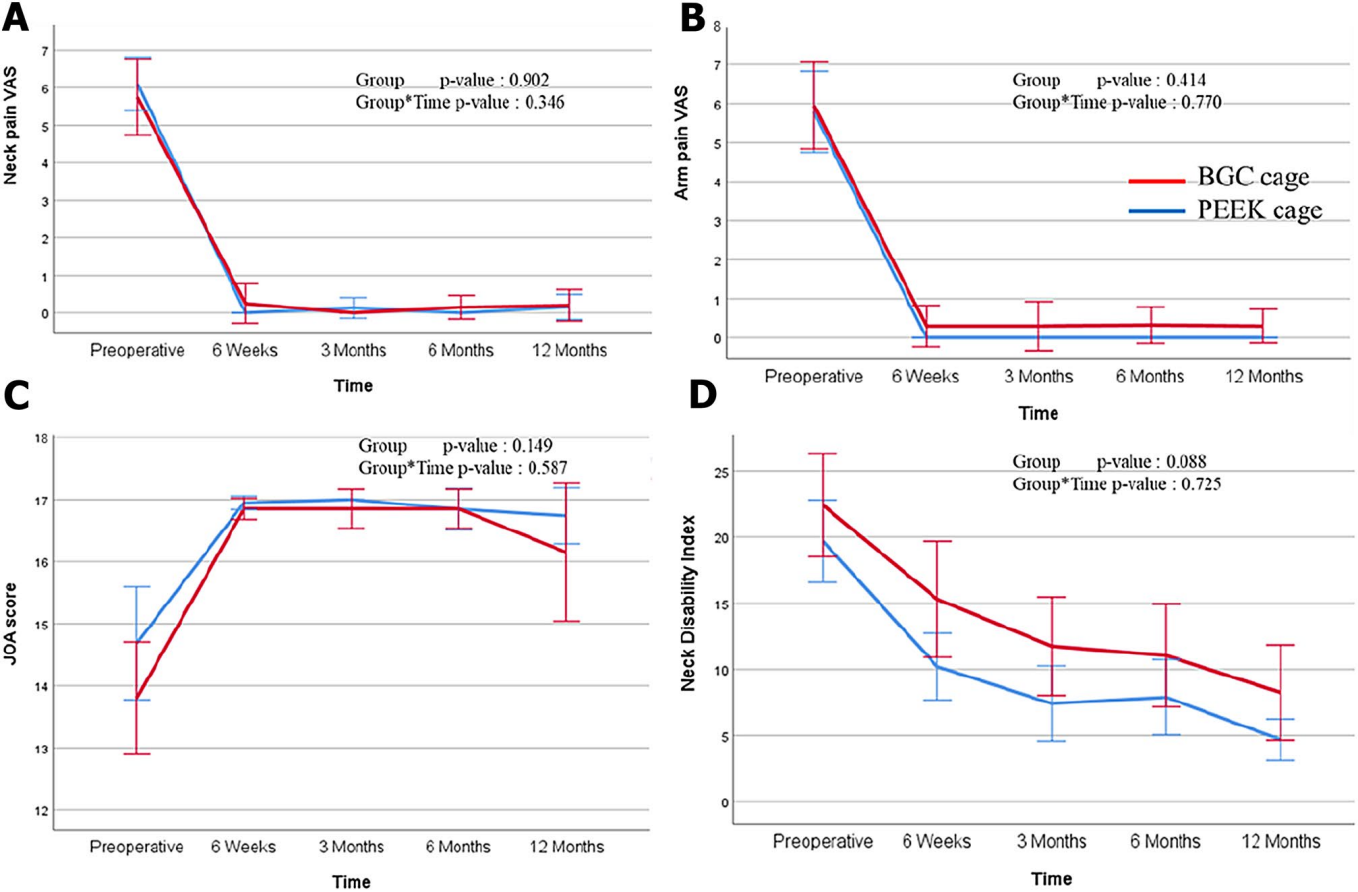
Clinical visual analogue scale scores of neck pain (**A**) and arm pain (**B**), Japanese Orthopedic Association (JOA) score (**C**) and Neck Disability Index (**D**).

Demographic data, pre- and post-operative clinical outcomes, and radiological measurements were collected. Categorical variables, including sex, smoking, pseudoarthrosis, and fusion status, were identified in a CT scan, and evidence of subsidence was analyzed using the χ2 test. Continuous variables, such as age, ISM, and serial changes in pre- and post-operative clinical outcomes between the two groups, including neck or arm VAS, JOA, and NDI scores, were analyzed using a t-test. The threshold for statistical significance was set at *P* < 0.05. A figure was created using a linear mixed model to visualize the improvement of the clinical score (Fig. 4). The SPSS statistical software was used in data analysis (SPSS, Inc., Chicago, Illinois, USA).

## Results

### Demographic data

Overall, 39 patients were recruited: 19 and 20 patients in the PEEK and BGC groups, respectively). However, one patient in the PEEK group was lost in the final follow-up (12 months post-operatively). The mean ages were 47.37 ± 13.5 and 50.55 ± 14.9 years in the PEEK and BGC groups, respectively (*P* = 0.536). Demographic data did not show a substantial difference between the PEEK and BGC groups. The PEEK and BGC groups had five and seven smokers, respectively, without significant differences (*P* = 0.55). C5-6 was the most common level of the operation (PEEK group: 13/19 [68.4%], BGC group: 16/20 [80%]). Regarding the patients’ pre-operative diagnosis, herniated cervical disc-induced radiculopathy symptoms accounted for approximately 60% of the two groups, followed by cervical spondylotic myelopathy and combined diagnosis, without significant difference (*P* = 0.873) (Table 1).

**Table 1:** Demographic data between patients with PEEK and BGC groups.

	PEEK group (n = 19)	BGC group (n = 20)	*P*-value
Age (years)	47.37 ± 13.5	50.55 ± 14.9	0.536
Sex (Female/Male)	9/10	11/8	0.634
BMI (kg/m^2^)	24.47 ± 3.35	23.93 ± 2.68	0.559
Current smoking (n)	5 (26.3%)	7 (35.0%)	0.557
Preoperative diagnosis			0.873
Herniated cervical disc	11 (57.9%)	12 (60.0%)	
Cervical spondylotic myelopathy	5 (26.3%)	4 (20.0%)	
Combined	3 (15.8%)	4 (20.0%)	
Treated spine level			
C3-4	2	0	
C4-5	0	1	
C5-6	13	16	
C6-7	4	2	
Coexisting medical condition			
Hypertension	5 (26.3%)	4 (20.0%)	0.640
Diabetes mellitus	0 (0.0%)	1 (5.0%)	0.323
Thyroid disease	0	0	
COPD or pneumonia	0	0	
Congestive cardiac failure	0	0	
Peripheral vascular disease	0	0	
Any solid tumor	0	0	

Values are expressed as mean ± standard deviation or percentages. Continuous variables, including age and BMI, were analyzed using a t-test. Categorical variables, including sex, smoking, diagnosis, treated level, and medical conditions, were analyzed using the χ^2^ test.

*BMI* bone mass index, *COPD* chronic obstructive pulmonary disease.

### Clinical outcomes

Pre-operative scores, including neck or arm pain Visual Analog Scale (VAS), Japanese Orthopedic Association (JOA), and Neck Disability Index (NDI) scores, did not substantially differ between the two groups. The NDI score was 10.21 ± 5.22 and 15.3 ± 9.36 in the PEEK and BGC groups, respectively, at 6 weeks post-operatively, without significant difference (*P* = 0.076). The trend of change in JOA score post-operatively was similar and showed no substantial difference. Comprehensively, no substantial difference was found in all post-operative serial scores (neck and arm VAS, JOA, and NDI scores) between the PEEK and BGC groups. Furthermore, a substantial improvement was noted in the post-operative serial scores compared to the pre-operative baseline score in the two groups (Fig. 4).

### Radiological outcomes

Inter-spinous motion (ISM) between fusion segments 12 months post-operatively in the PEEK and BGC groups was 0.54 ± 0.37 and 0.54 ± 0.37 mm, respectively (*P* = 0.562). No subsidence occurred at 12 months post-operatively in the two groups. However, one case in each group showed pseudoarthrosis in the dynamic radiograph (PEEK group: 1.6 mm, BGC group: 1.3 mm), and no evidence of bone bridging formation was noted around the cage between fusion segments in the CT scan at 12 months post-operatively (Table 2). The Kappa value for inter-observer reliability was 0.62, indicating substantial agreement, whereas those for intra-observer reliability were 0.620 and 0.661 for each measurement.

**Table 2:** Surgery data between patients with PEEK and BGC group.

	PEEK group (n = 19)	BGC group (n = 20)	*P*-value
Operative time (min)	105.57 ± 14.80	75.80 ± 16.05	< 0.001
Intraoperative blood loss (mL)	75.26 ± 75.74	58.25 ± 53.29	0.42
Blood transfusion (number of patients)	0	0	
Inter-spinous motion at post-operative 1 year (mm)	0.54 ± 0.37	0.54 ± 0.37	0.562
Bone bridging formation at post-operative 1 year (Y/N)	18/19 (94.7%)	19/20 (95.0%)	0.970
Causes of intra-operative/post-operative adverse events			
Dural tear (n)	0	0	
Post-operative neurologic deficit (n)	0	0	
Deep wound infection (n)	0	0	
Pseudoarthrosis (n)	1 (5.3%)	1 (5.0%)	0.970
Subsidence (n)	0	0	
Reoperation due to perioperative adverse events	0	0	
Readmission due to perioperative adverse events	0	0	

Values are expressed as number of patients or percentages. Continuous variables, including operative time, blood loss, and inter-spinous motion, were analyzed using a t-test. Categorical variables, including blood transfusion, bone bridging formation, adverse events, reoperation, and readmission, were analyzed using the χ^2^ test.

### Surgical results and adverse events

Operative time was significantly shorter in the BGC group than in the PEEK group (PEEK vs. BGC group: 105.57 ± 14.80 vs. 75.80 ± 16.05 min, *P* < 0.001), although intra-operative blood loss did not significantly differ (PEEK vs. BGC group: 75.26 ± 75.74 vs. 58.25 ± 53.29 mL, *P* = 0.42). No unexpected adverse events, including dysphagia, hematoma, surgical site infection, dural tear, and re-operation, were found in this study (Table 2). In the PEEK group, possible donor site morbidities, such as pain, infection, hematoma, seroma, and meralgia paresthetica, after iliac bone harvest were not reported during this study.

## Discussion

In this study, BGC showed no substantial difference with PEEK cage with autologous iliac bone graft. Both groups showed favorable fusion rates and improvement in clinical and radiological outcomes. Operative time was shorter in the BGC group than in the PEEK group because harvesting autologous iliac bone can be skipped in the BGC group. An additional incision is generally added when a bone graft is performed; therefore, bleeding, seroma, post-operative fracture, infection, and post-operative pain may have occurred^5,6^. Contrary to the author’s expectation, intra-operative blood loss showed no considerable difference. Since the bone graft technique was performed using minimally invasive surgery, no substantial difference was found in the bleeding count, and no difference in blood transfusion was observed between the two groups. The donor site morbidity-associated score was not measured in the clinical outcome-related questionnaire used in this study. However, in a subset of JOA, scores related to lower extremity motor functions were found, but without substantial difference between the two groups. In the linear mixed analysis of this study’s clinical outcome (Fig. 4C), examining whether the high recovery rate of the JOA score in the BGC cage compared to the PEEK cage is not because of one donor site morbidity will be necessary. However, it should be noted that the difference was not substantial 6 weeks post-operatively. Therefore, this suggests that the difference in clinical scores is not substantial unless donor-site morbidity-related complications occur^6^.

Various graft materials have been available since ACDF was introduced in the 1950s^9,10^. Autologous iliac bone graft and fibular strut were the first generally accepted graft materials. An increased risk of donor site morbidities, such as pain or infection, is linked to the widespread usage of auto-graft material. A review of the literature has revealed various graft materials that can be used to overcome donor site morbidity^5^. However, surgeons are always concerned about these materials since they have certain issues^25^. Allografts (mostly freeze-dried grafts from cadaveric bone) and xenografts (from an animal) have been used with satisfactory outcomes; however, concerns exist about pseudoarthrosis, immuno-compatibility issues, and the risk of transmissible diseases^26^. Synthetic alternatives to autologous or allogenic grafts have been developed to prevent harvesting complications, increase fusion rates, and improve clinical outcomes^27^. Cages or interbody fusion devices have been developed for substitutes, including carbon fiber reinforced by polymers, titanium, and PEEK, with PEEK being the most commonly used graft among them^28^. Titanium cage has a different elastic modulus, making it prone to penetrating the vertebral endplate, which may result in subsidence; additionally, its metallic artifact in magnetic resonance imaging (MRI) can pose challenges in the assessment during post-operative evaluation^29^. Park et al. reported that a BGC cage resulted in less artifact generation in MRI compared to a titanium cage^13^. Titanium cages have been reported to show a relatively high subsidence rate due to their stronger physical properties than PEEK cages^30^. PEEK cages with local bone grafts have prevailed for higher fusion rates and favorable outcomes. However, some authors suggested that the fibrous layer in a bone-PEEK cage can make poor integration with host bone in sheep tibia model^31^. Prior to BGC, ACDF was explored using a ceramic spacer made of hydroxyapatite (HA) and tricalcium phosphate (TCP). Ceramic spacers, including HA, are known to have osteoconductivity due to their porous structure. Zadegan et al. reviewed various ceramic cages and concluded that the solitary use of ceramic spacers is associated with graft fracture and crack. This suggests that the ceramic spacer has an osteoconduction effect because of its porous structure; however, it simultaneously should have physical properties that can withstand graft fracture or cracks^9^. Lee et al. introduced a derivative of BGC-formed CaO–SiO_2_–P_2_O_5_–B_2_O_3_, known as BGS-7, which has approximately 13 times more compressive strength and 2 times more bending strength than human compact bone and simultaneously has a porous structure, with the property of inducing osteoconduction^32^.

In this study, no difference was found in the fusion rate and subsidence between the PEEK and BGC groups on the radiological results, including ISM and computed tomography (CT) evaluation, although no additional autologous bone graft from the iliac bone was found in the BGC group. One case of pseudoarthrosis confirmed by radiographic ISM was found in each group, and no subsidence demonstrated by CT evaluation was found in either group. The incidence of subsidence and pseudoarthrosis in the PEEK cage is reportedly up to approximately 35% and 52%, respectively, although they differ between studies^33^. However, relatively favorable outcomes were obtained in our study. Therefore, it can be assumed that adjusting the local morselized bone from the resected osteophyte and bone dust surrounding the graft and the osteoconducting characteristics of BGC produced results similar to those of the PEEK cage with autologous iliac bone^34^. Additionally, the absence of mechanical complications, such as cage breakage, can be attributed to the superior mechanical strength of the BGS cage compared to the PEEK cage. Based on the BGC cage’s external characteristics, the reason for the excellent fusion rate can be inferred as follows. According to Fig. 1, the BGC cage used in this study has no cage window inside. The cage in ACDF initially serves as a scaffold to support vertebral bodies requiring union. Additionally, the 15 × 13 × 7-mm sized BGC cage used in this study has a contact area of 216.93 mm^2^, whereas the PEEK cage has a considerably smaller contact area of 127.64 mm^2^ due to the window. This has important implications in which more graft-body interfaces in mechanical properties can lead to more stable scaffold outcomes. The BGC material, which has excellent scaffold properties and a porous structure favorable for osteoconduction, is superior to the PEEK cage, which has a bone substitute that can induce osteoconduction added to the cage window. In the clinical outcomes, both groups showed substantial improvement regarding neck and arm pain VAS, JOA, and NDI compared with the pre-operative status, without substantial intergroup and time differences. Additionally, no difference was found between the two groups in the incidence of surgery-related complications, such as dural tears, post-operative neurologic deficit, and deep wound infection. No difference was found in intra-operative blood loss or resulting blood transfusion. Therefore, it can be noted that the BGC cage does not affect the routine procedure conducted by the surgeon when performing one level ACDF.

Some authors reported superior clinical and radiological outcomes in structural allobone cages compared to PEEK cages^35,36^. In North America, the use of structural allobone cages has become popular in ACDF; however, tri-cortical autologous iliac bone graft or PEEK cages are widely used worldwide. PEEK cage is a commonly used synthetic material among various options for ACDF. It also exhibits an elastic modulus similar to that of the human bone, resulting in reduced cage subsidence and improved load distribution between the cage and bone. The characteristics of radiolucency on plain X-ray and CT scans are relatively familiar and convenient when assessing the status of fusion and reducing the impact of implant artifacts on post-operative CT or MRI scans^11^. In a consensus survey for ACDF graft selection by surgeons reported in 2017, PEEK cages were reported as the graft of choice in overall global demographic data, followed by autologous bone and allogenic grafts as the most selected, although allogenic bone graft is most commonly used by north American surgeons^37^. However, the PEEK cage lacks the necessary biological properties for promoting bony fusion, namely osteoconduction and osteoinduction. Therefore, PEEK cages have been augmented with various substances, including autologous local bone, HA, β-TCP, and DBM, to address this constraint^10^. However, these substances incur a separate cost. When considering the cost-effectiveness aspect, accounting for the cost of the graft is also important^38^. Synthetic ceramic cage showed the lowest cost, PEEK was followed by allograft showed the highest cost. Therefore, the BGC cage can be a good alternative in the medical reality of patients with insufficient income or in regions where using the relatively expensive allobone cage is burdensome. However, further comparative studies with previous cages are still needed. Additionally, a comparative study with a three-dimensional titanium cage with a porous structure, which has recently been introduced and attempted, is also needed. To our knowledge, this is the first comparison study with a PEEK cage filled with autologous bone and BGC. Recently, a comparative study was conducted using PEEK cages with hydroxyapatite, TCP, and BGC cages. However, since the previous study did not compare using autologous bone, which can be considered a standard substitute for bone induction, it can be noted that a lack of clinical comparison including bony union may exist^11^. Therefore, follow-up studies supporting the safety and effectiveness of BGC are needed. Furthermore, if favorable outcomes are shown in the comparison of multilevel ACDF and long-term follow-up in the future, BGC can be a worthy alternative based on cost-effectiveness, safety, and clinical effectiveness.

This study had some limitations. First, it had a limited sample size, short-term follow-up, and retrospective design. Therefore, longer follow-up data with larger case series are needed to clarify these results. Second, the BGC or PEEK cage was selected to ensure that the surgeon’s preference was not affected by dividing it into a specific period. Therefore, this study did not proceed with randomization for the PEEK and BGC groups, and the possibility of bias due to the surgeon's preference cannot be ruled out. Third, ACDF could not be performed alone in a cage in this study. The additional anterior cervical plate system can act as a variable for a more accurate comparison between the PEEK and BGC cages^39^. In this study, the same anterior cervical plate system (VENTURE^®^, Medtronic, MN, USA) was used for both procedures to minimize this bias. Therefore, it appears that the mechanical properties within the cage for different types of loads of compression shear, torsion, subsidence, and expulsion may vary to some extent depending on the anterior cervical plate. Besides, complications, such as adjacent segment degeneration, could not be investigated because long-term follow-up was not performed, and this should be revealed through additional research in the future.

## Conclusion

This study shows that non-window-type BGC cage is an effective alternative for spacer use in single-level ACDF with favorable outcomes, and no substantial difference was observed in clinical performance with PEEK cage with autologous iliac bone graft.

## Data Availability

The data that support the findings of this study are available from the corresponding author, [Kyung Soo Suk], upon reasonable request.
